# A pilot study to compare propranolol and misoprostol versus misoprostol and placebo for induction of labor in primigravidae; a randomized, single-blinded, placebo-controlled trial

**DOI:** 10.1186/s12884-023-05537-1

**Published:** 2023-04-04

**Authors:** Ahmed Sherif Abdel Hamid, Hazem El Zeneiny, Ahmed Fathy, Maii Nawara

**Affiliations:** Department of Obstetrics & Gynecology, Faculty of Medicine, Ain Shams University, Abbaseyya Square, Cairo, Egypt

**Keywords:** Propranolol, Misoprostol, Induction of labor

## Abstract

**Background:**

The Induction of labor is the most common obstetric procedure in daily practice. Introducing propranolol as a new drug to augment the action of prostaglandins will help in the induction process and decrease CS rates. Several researchers have used propranolol in the augmentation of labor.

**Aim:**

This pilot study compares propranolol and misoprostol versus misoprostol alone for labor induction in primigravids.

**Methods:**

This is a Randomized clinical trial, single-blinded, placebo-controlled trial at Ain Shams University Maternity hospital. This study included 128 pregnant full-term primigravid women candidates for labor induction, randomized into two groups. All candidates underwent labor induction with 25 µg of vaginal misoprostol. Group I received 20 mg of oral propranolol tablets, while group II received sugary pills as a placebo. Candidates who responded successfully to induction were assessed for possible augmentation of labor by amniotomy or oxytocin infusion. The Primary outcome was induction to delivery interval, while the secondary outcomes were the duration of the latent phase, mode of delivery, and APGAR score of the neonate.

**Results:**

The induction-delivery time was (11.8 ± 8.1 h. vs. 12.6 ± 8.9 h., P value = 0.027) and the duration of the latent phase of labor (7.9 ± 5.6 h. vs. 9.2 ± 6.03 h., P value = 0.017) were significantly shorter in the group of misoprostol and propranolol compared to the group of misoprostol and placebo. There was no statistically significant difference between both groups’ mode of delivery, indications for cesarean section, misoprostol, and oxytocin doses, or neonatal outcome. (P value > 0.05).

**Conclusion:**

Propranolol, when used with misoprostol for induction of labor, results in augmentation of action of misoprostol and a significantly shorter induction-delivery interval.

**Trial registration:**

We retrospectively registered this trial in clinicaltrial.gov on 01/09/2020 (NCT04533841). https://clinicaltrials.gov/ct2/show/NCT04533841

## Synopsis

propranolol augments the action of misoprostol in the induction of labor causing shorter induction to delivery time.

## Introduction

Induction of labor at or beyond 37 weeks of gestation is associated with a clear reduction in perinatal death compared to expectant management. It also reduces cesarean section rates without increasing operative vaginal deliveries and fewer Neonatal Intensive Care Unit (NICU) admissions [1]. Generally, labor induction is indicated when the risks of pregnancy continuation are thought to outweigh its benefits [2]. In the UK, labor inductions increased from 29.4% in 2016–2017 to 31.6% in 2017–2018, [3] and they constitute 24.5% of vaginal deliveries in the USA [4].

However, induction failure was reported as high as 23.7% in multiparas and 41.2% in nulliparas [5]. In Egypt, the rates of inductions vary widely depending on private/government facilities, nonteaching, and teaching(tertiary) care hospitals. Failure of induction is one of the causes of high rates of CS in Egypt that reached (54%) of total deliveries [6].

These facts explain why every effort is made to shorten the induction to the delivery interval with the least maternal and neonatal morbidities. Induction of labor in circumstances where the cervix is unripe with a low Bishop score carries a higher chance of failure [7]. Various methods are used to prepare the cervix for labor including; mechanical and pharmacological methods, such as prostaglandins or oxytocin [8].

Misoprostol, a synthetic PGE1, has uterotonic properties, by contracting smooth muscle fibers in the myometrium and cervical relaxation, facilitating cervical dilatation and effacement. Compared to other prostaglandins, misoprostol has several potential advantages. It is stable at room temperature, cheap, and can be given by several routes [9].

Propranolol is a non-selective beta blocker that was studied to induce uterine contractions in several studies. This triggered a wide investigation of the presence of beta receptors in human myometrium and their role in myometrial contractility [10–13]. This study aims to determine if propranolol might shorten the induction-delivery interval when added to misoprostol.

## Materials and methods

This randomized controlled parallel arm trial has a 1:1 allocation for each arm (propranolol/misoprostol is the active arm and the placebo/misoprostol is the control arm). The study was carried out on 128 women in Ain Shams university Maternity hospital, who were candidates for induction of labor in the period between February 2020 and OCTOBER 2020. Before the beginning of the study, the protocol gained the Scientific and Research approval from the council of the OB/GYN Department, Ain-Shams University. Furthermore, the study protocol was approved by the Ethics Research Committee, Faculty of Medicine, Ain-Shams University (number: FMASU M S 410 / 2020). Written informed consent was obtained from every candidate after explaining the procedure before enrollment. All methods were performed in accordance with the relevant guidelines and regulations (Declaration of Helsinki), then the study was registered at clinicaltrials.gov on 01/09/2020 (NCT04533841). No Important changes were done to methods after trial commencement.

The number of patients assessed for eligibility was 163. The eligibility criteria included full-term primigravid women, with singleton pregnancies, cephalic presentation, Bishop score < 5, ultrasound-confirmed gestational age dates, and normal fetal heart rates. The exclusion criteria; women presented in active labor, had suspected fetal macrosomia, polyhydramnios, showed any signs of fetal distress, gave a history of previous uterine surgery, hypersensitivity to prostaglandins, asthma, liver or kidney impairment, a known cardiac disease with abnormal ECG, or had any obstetric contraindication to labor induction such as active genital herpes or placenta previa.

Study candidates underwent full history taking, and general, abdominal, and pelvic examinations. Candidates were randomized into two groups, 64 women in each. Group I received 2 tablets of propranolol Hydrochloride 10 mg (Indral, Astrazinca Egypt), while group II received 2 sugary tablets in the same size, color, and shape as the propranolol tablets, 30 min after the start of the induction process. Labor induction was performed by inserting 25 µg of vaginal misoprostol (Vagiprost, Adwia, Egypt) every 6 h till achieving 3–5 uterine contractions each lasting 30–50 s, and adequate cervical ripening has been reached (Bishop score > 7), spontaneous rupture of membranes or 24 h have elapsed (classified as failed induction). Candidates who responded successfully to induction (by achieving at least 3 strong uterine contractions lasting 30–50 s) were assessed for possible augmentation of labor by amniotomy or oxytocin infusion. After induction, women were monitored for fetal and maternal well-being and labor progress.

**The Primary outcome was the** induction-delivery interval while the **secondary outcomes were the** duration of the latent phase, mode of delivery, and APGAR score of the neonate.

No changes to trial outcomes occurred after the trial commenced.

Regarding **the sample size calculation**, there was no adequate information in the literature comparing misoprostol alone with misoprostol and propranolol; the present exploratory study targets a clinically significant effect size. It was estimated that a sample size of 64 women in each group (total 128) will achieve a power of 80% to detect statistically significant differences between the two groups regarding qualitative variables for a median effect size corresponding to a cohen’s d coefficient of 0.5 with α- error 0.05 using G-power software for sample size calculation.

Candidates were randomized into two groups. The randomization sequence was created using computer-generated randomization with a 1:1 allocation for each arm of the study. Each candidate was assigned an opaque sealed envelope with her number containing either propranolol tablets or a placebo. Both the patient and doctor were blinded to which group the patient was assigned to, only known by the nurse in the outpatient clinic where she chose each envelope for each patient.

The collected data was revised, coded, tabulated, and introduced to a PC using the Statistical Package for Social Science (SPSS, Version 20.0). Shapiro Wilk’s test was used to evaluate the normal distribution of Quantitative variables. The tests used were Student’s t-test (for numeric parametric variables), Mann-Whitney’s U test (for numeric non-parametric variables), and chi-squared or Fisher exact test (for categorical variables) as indicated. A P-value < 0.05 was considered statistically significant.

## Results

**Fig. 1:**
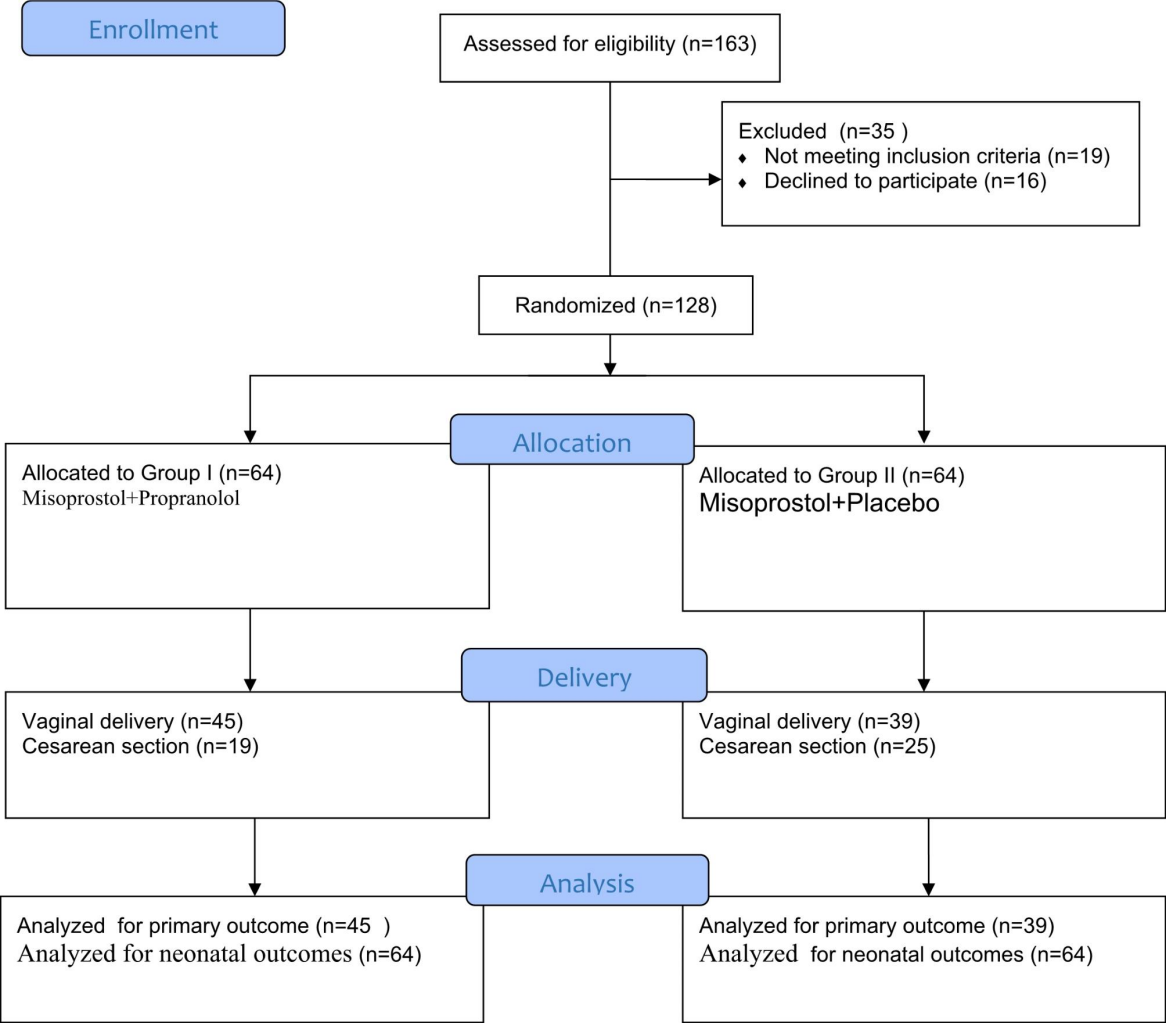
Recruitment and flow of patients with induction of labor

The number of patients enrolled in the study was 163, The number of excluded women is 35; 16 patients declined to participate and 19 patients were excluded as they did not meet inclusion criteria. We randomized 128 women into two equal groups as shown in the CONSORT flow diagram (Fig. 1) during the period from February 2020 and October 2020.

Table 1 shows no statistically significant differences between both groups regarding age, Body Mass Index (BMI), gestational age, and Bishop score at admission.

**Table 1:** Personal and obstetric characteristics of both groups

		Group I Propranolol &misoprostol (N = 64)	Group II placebo &misoprostol (N = 64)	P-value
**Age (years)**	**Mean ± SD**	23.3 ± 4.1	22.9 ± 3.2	0.533 NS^a^
**BMI (kg/m** ^**2**^ **)**	**Mean ± SD**	26.4 ± 2.7	25.7 ± 2.7	0.141 NS^a^
**Bishop score**	**Mean ± SD**	4.1 ± 1.08	3.8 ± 1.2	0.094 NS^b^
**G. Age (weeks)**	**Mean ± SD**	41.04 ± 1.3	41.2 ± 1.3	0.549* NS^a^

^a^Student t-test

^b^Mann-Whitney test

*NS* Non-significant, *N* number, *BMI* Body Mass Index

The indication of induction in both groups was as follows; post-date (60.9% in group 1 vs. 68.8% in group 2), ROM (28.1% in group 1 vs. 15.6%in group 2), HTN (9.4% in group 1 vs. 14.1%in group 2), and GDM (1.6% in group 1 and 1.6% in group 2). Table 2 shows a higher proportion of cesarean sections in Group II than in Group I (39.1% vs. 29.7%), yet this difference failed to reach statistical significance. There were significant differences between both groups regarding indications for cesarean section. The 45 women in Group I and the 39 women in Group II who delivered vaginally were statistically analyzed for the duration of labor, and total doses of misoprostol and oxytocin received. Table 2 shows a significantly shorter latent phase of labor in Group I when compared to Group II (7.9 ± 5.6 h. vs. 9.2 ± 6.03 h.), as well as a significantly shorter induction-delivery interval (11.8 ± 8.1 h. vs. 12.6 ± 8.9 h.). There was no significant difference between both groups regarding the duration of the active phase of labor. Both groups had no significant differences regarding the total number of misoprostol doses to achieve successful induction or the total doses of oxytocin to maintain adequate uterine contractions as shown in Table 2. There were no reported side effects of propranolol in group I as headache, dizziness, nausea, or vomiting.

**Table 2:** Labor characteristics of both groups in both groups

		Group I Propranolol &misoprostol N = 64		Group II placebo &misoprostol N = 64		P-value		
**Indication of induction**	**Post date (n %)**	39	60.9%	44	68.8%	0.355 NS 1		
	**ROM (n %)**	18	28.1%	10	15.6%	0.08 NS 1		
	**HTN (n %)**	6	9.4%	9	14.1%	0.41^*^NS 1		
	**GDM (n %)**	1	1.6%	1	1.6%	1.0 NS 2		
**Mode of delivery**	**NVD (n %)**	45	70.3%	39	60.9%	0.264 NS 2		
	**CS (n %)**	19	29.7%	25	39.1%			
**Duration of Latent phase (hours)** **(45 cases in group I**, **39 cases in group II)**	**Mean ± SD**	7.9 ± 5.6		9.2 ± 6.03		**0.017 S 3**		
**Duration of Active** **Phase (hours)** **(45 cases in group I**, **39 cases in group II)**	**Mean ± SD**	3.9 ± 2.7		3.5 ± 2.9		0.425 NS 3		
**Induction-delivery interval (hours)** **(45 cases in group I**, **39 cases in group II)**	**Mean ± SD**	11.8 ± 8.1		12.6 ± 8.9		**0.027 S 3**		
**Misoprostol dose (No. of doses) (45 cases in group I, 39 cases in group II)**	**1 dose (n %)**	14	31.1%	14	35.9%	0.79 NS 2		
	**2 dose (n %)**	28	62.2%	22	56.4%			
	**3 dose (n %)**	2	4.4%	2	5.1%			
	**4 dose (n %)**	1	2.2%	1	2.6%			
**Oxytocin dose(units)** **(45 cases in group I, 39 cases in group II)**	**0 unit (n %)**	26	57.8%	22	56.4%	0.834 2 NS		
	**5 units (n %)**	14	31.1%	13	33.3%			
	**10 units (n %)**	4	8.9%	2	5.1%			
	**15 units (n %)**	1	2.2%	2	5.1%			
**Indication of CS** **(19 cases in group I, 25 cases in group II)**	**Failed progress (n %)**	7	36.8%	9	36%	0.713 NS 2		
	**Fetal distress (n) %)**	10	52.6%	15	60%			
	**Failed induction (n %)**	2	10.5%	1	4%			

^a^Chi square test

^b^Fisher exact

^c^Mann Whitney test

*S* significant, *NS* Non-significant, *ROM* Rupture of membranes, *HTN* Hypertension, *GDM* Gestational Diabetes Mellites, *NVD* Normal Vaginal Delivery, *n* number

The neonatal outcome of all the women participating in the study was analyzed to detect any adverse effects caused by propranolol given before labor. There were no statistically significant differences between both groups regarding APGAR scores at 1 and 5 min and the need for neonatal intensive care unit (NICU) admission as shown in Table 3.

**Table 3:** Neonatal outcome in both groups

		Group I Propranolol &misoprostol n = 64		Group II placebo &misoprostol n = 64		P-value
**Fetal weight (kg)**	**Mean ± SD**	3.45 ± 0.4		3.53 ± 0.4		0.229 NS^a^
	**Median**	3.5		3.6		
**APGAR score** **1 min** **APGAR below 7**	**Mean ± SD**	7.5 ± 0.9		7.45 ± 1		0.798 NS^a^
	**Median**	8		8		
		10 (15.62%)		9 (14.06%)		
**APGAR score** **5 min** **APGAR below 7**	**Mean ± SD**	8.9 ± 0.6		8.8 ± 0.6		0.312 NS^a^
	**Median**	9		9		
		1 (1.56%)		1 (1.56%)		
**NICU**	**No**	61	95.3%	58	90.6%	0.49 NS^b^
	**Yes**	3	4.7%	6	9.4%	

^a^Mann Whitney test

^b^Fisher exact test

*NS* Non-significant, *n* number, *NICU* Neonatal Intensive Care Unit

## Discussion

In the present study, there was no statistically significant difference between the 2 groups regarding age, BMI, gestational age, or Bishop score in all induced ladies. The most common indication of induction in the present study was postdated pregnancy, followed by PROM. Our study found that adding propranolol to misoprostol during induction of labor results in a significantly shorter induction-delivery interval and a significantly shorter latent phase of labor when compared to misoprostol alone. Adding propranolol ended in fewer cesarean sections, yet this difference failed to reach statistical significance, with no significant differences regarding indications for cesarean section. The rate of CS in the present study was 29.7% which is lower than higher rates in Egypt which reached 54% of total deliveries. Our hospital is the biggest tertiary university hospital in Cairo governorate with more than 20,000 deliveries per year. Patients who received propranolol did not require significantly lower doses of misoprostol for induction or oxytocin for augmentation of labor. There was no significant difference in neonatal outcome between those who received propranolol with oxytocin and those who received oxytocin only.

The beta-2 adrenergic receptor, a member of the family of G protein-coupled receptors is widely expressed in the uterus. Activation of this receptor is important in smooth muscle relaxation resulting from signaling the adenylate cyclase pathway. Propranolol is a β-adrenergic receptor–blocking agent that has been shown and demonstrated to increase uterine activity among pregnant females acting by withdrawing the suppressive impact of the β-agonist isoproterenol on uterine motility in humans [14].

### Comparison of our results to related studies

Different studies handled propranolol in different aspects of labor beginning early with dysfunction of labor, induction of labor by IV propranolol with oxytocin, the active stage of labor with oxytocin, oral propranolol, and oxytocin with induction, a recent study only with misoprostol but with IV propranolol, our study compares oral propranolol and vaginal misoprostol in labor induction.

The first uncontrolled trial studying the effect of propranolol on dysfunctional labor was conducted in 1975 by Mitrani and colleagues. IV propranolol was administered to ten primigravidae with dysfunctional labor. This was followed by normal uterine activity and delivery without significant maternal or fetal complications. They assumed that labor pains were accompanied by fear and anxiety due to the release of catecholamines, which activated beta-adrenergic receptors causing weak uterine contractions [15].

In the study of Sanchez-Ramos et al., 1999 regarding parturients with dysfunctional labor, ninety-six parturients were randomized during the active stage of labor to propranolol 2 mg IV versus placebo with continuous oxytocin infusion. Their results showed significant improvement in uterine contractility and a subsequent decrease in CS rates [16].

The effect of IV propranolol/oxytocin in labor induction vs. placebo/oxytocin was handled in several studies. In the double-blind randomized controlled trial of Kashanian et al., 2008, 150 nulliparas parturients were randomized into 2 groups. In the first group oxytocin alone was used for the induction of labor. In the second group, 2 mg propranolol was slowly injected intravenously then the oxytocin was initiated. In agreement with our study, their results showed a shorter induction delivery time and shorter latent phase of labor. Contrary to our study, the amount of necessary oxytocin used was significantly less in the propranolol group [17].

In agreement with our study is the meta-analysis by Pergialiotis et al. 2016 on the effect of adding propranolol with oxytocin during the latent and active phases of labor. They enrolled 609 parturients from 6 studies. They concluded that there is evidence supporting that propranolol shortens the latent phase, possibly the Induction-delivery time, and has no effect in decreasing the active stage of labor. Contrary to our study, they concluded that propranolol effectively lowered the cesarean section rates (OR 0.49, 95% CI 0.27, 0.89). This meta-analysis also showed no difference in the 5 min neonatal Apgar scores and NICU admissions, which agrees with the present study [18].

Oral Propranolol was used in the study of Moghadam et al., 2013 in which 146 nulliparous women undergoing labor induction were randomized into 2 groups; the first group used propranolol administered orally in a dose of 20 mg then introduced oxytocin, and the second group placebo was taken before oxytocin. In agreement with our results, they showed a significantly shorter induction-delivery time and shorter latent phase of labor. Contrary to our study, they found significantly lower cesarean section rates in the propranolol group [19].

The study of Bigelow et al., published in 2020, was the same as our study design; it was a single-blind, randomized, placebo-controlled trial of Primigravidae patients undergoing labor induction either. Two hundred and Forty Patients were randomized to receive 2 mg of IV propranolol or saline placebo before induction by 30 min. There were 240 patients enrolled in this study with 121 patients randomized to propranolol and 119 to placebo. About 64.2% of patients (154) delivered vaginally. There was no statistically significant difference in the induction-delivery time as a primary outcome. There was no statistically significant difference in the secondary outcomes (in cesarean section rate, time to active labor, or time to full dilatation in patients receiving propranolol vs. placebo. Interestingly they found a significantly lower rate of composite maternal morbidity in the propranolol group with lower rates of postpartum hemorrhage and transfusion [20].

The discrepancies between our results and the results of previous studies may be attributed to the fact that we used misoprostol for induction of labor, unlike all the other studies, and that we used oral propranolol which has a longer lasting half-life compared to the intravenous propranolol used in most of the previous studies. Despite all the discrepancies, propranolol has been shown to affect myometrial contractility and the course of labor with no significant neonatal effects.

### Clinical implication of our study

The results of our study support the positive role of oral propranolol in the induction of labor. Propranolol shortened the induction-delivery interval when added to misoprostol resulting in more success in labor induction and lower rates of Caesarean sections. Propranolol is a cheap drug with minimal side effects; we think it could be used as an adjuvant to misoprostol in labor induction.

### Strengths and limitations of the present study

The strength of our study is that it is the first study to use oral propranolol and vaginal misoprostol in the induction of labor in PG patients.

In this study, we included women with prelabour rupture of membranes (PROM). We consider this a limitation to our study because (PROM) results in a shorter induction-to-delivery interval compared to intact membranes [21]. Nevertheless, their inclusion did not confound our results because there was no statistically significant difference between both groups regarding the indications for induction of labor (P = 0.08).

### Recommendations for further studies

Future research is needed to assess the role of propranolol in the induction process in multiparous women and the induction of patients with previous Caesarean sections.

## Conclusion

Propranolol, when used with misoprostol, for induction of labor results in augmentation of action of misoprostol and a significantly shorter induction-delivery interval so it can be used as an adjuvant to misoprostol.

## Data Availability

All Data and ethical committee documents are available from the corresponding author on reasonable request.

## Declarations

### Registration

The study was registered at clinicaltrials.gov on 01/09/2020 (NCT04533841).

## Abbreviations

CS: Cesarean Section
NICU: Neonatal Intensive Care Unit
PGE1: prostaglandin E1
